# Supplementary figures and images for: Growth Patterns and Scaling Laws Governing AIDS Epidemic in Brazilian Cities
Source: PLoS One. 2014 Oct 23;9(10):e111015. doi: 10.1371/journal.pone.0111015 (PMC4207789; doi:10.1371/journal.pone.0111015)

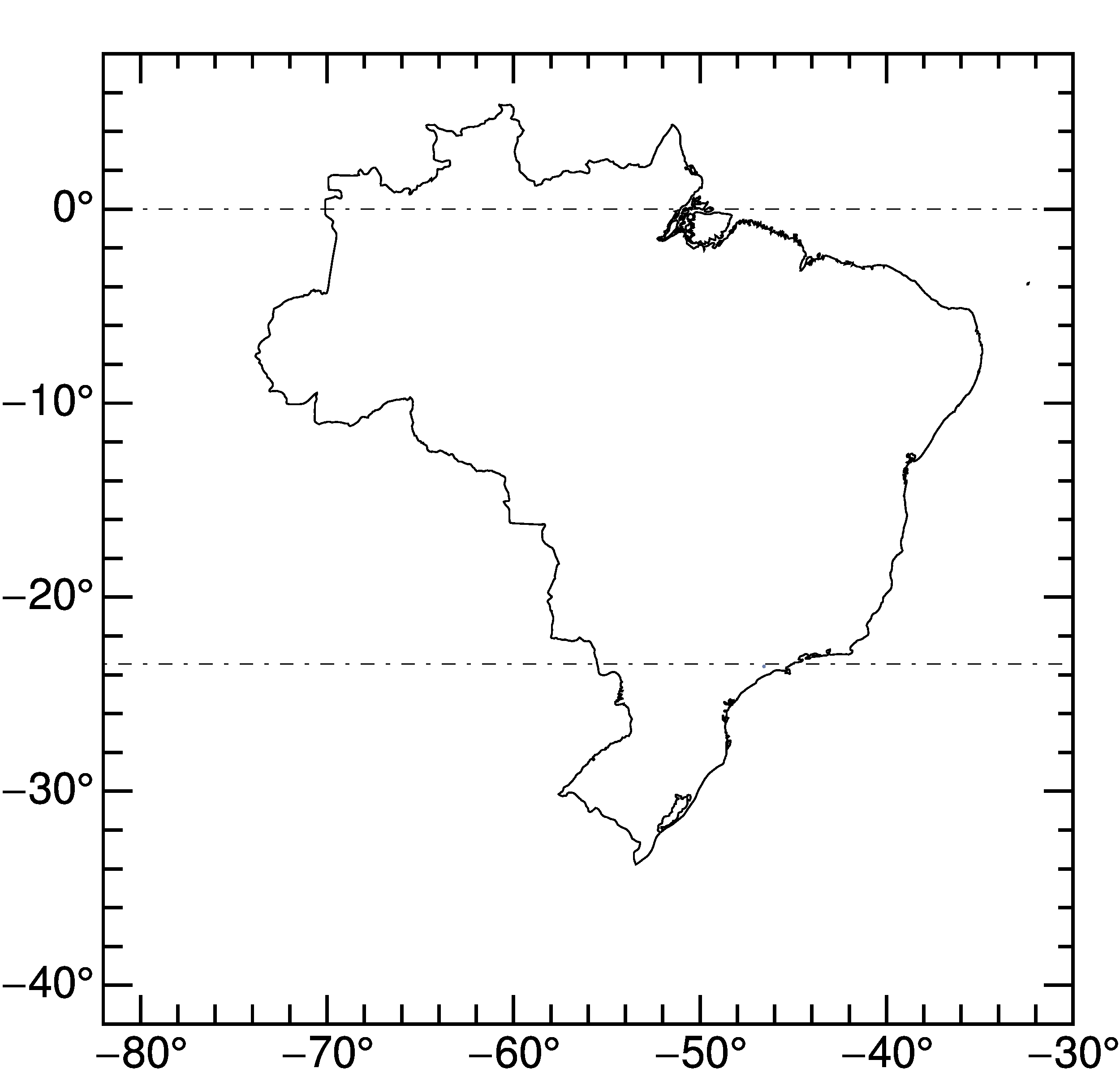

Supplement: Figure S1 — Chronological evolution of the AIDS epidemics among Brazilian cities. Besides reflecting in a great extent the population distribution, this animation also provides a general information concerning the spatial spreading of the epidemics over the country. (GIF) [file pone.0111015.s001.gif]
